# Recovery From Persistent Phrenic Nerve Palsy Following Pulsed Field Ablation: A Case Report

**DOI:** 10.1002/joa3.70523

**Published:** 2026-11-11

**Authors:** Tatsuya Hayashi, Shingo Yamamoto, Jumpei Ohashi, Midori Sato, Eiichi Shiraki, Yuya Watanabe, Yoshiki Asano, Kenichi Sakakura, Hideo Fujita

**Affiliations:** ^1^ Division of Cardiovascular Medicine, Saitama Medical Center Jichi Medical University Shimotsuke Japan

**Keywords:** catheter ablation, phrenic nerve palsy, pulsed field ablation

## Abstract

Persistent phrenic nerve palsy is a rare but possible complication of pulsed field ablation. We report recovery of phrenic nerve function approximately 6 months after PulseSelect ablation and discuss potential mechanisms and preventive strategies, including the effects of repeated applications and their spatial distribution around the right superior pulmonary vein.

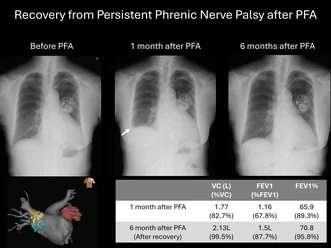

A 79‐year‐old woman with a history of pacemaker implantation for sick sinus syndrome was referred for catheter ablation of atrial fibrillation (AF). Her body mass index (BMI) was 24.1 kg/m^2^. Catheter ablation was performed using the PulseSelect pulsed field ablation (PFA) system (Medtronic, Minneapolis, MN), under general anesthesia with neuromuscular blockade. Bilateral pulmonary vein isolation (PVI) was successfully achieved without any apparent complications during the procedue (Figure 1). In the right superior pulmonary vein (RSPV), several PFA applications were additionally delivered within the vein to reduce the risk of pulmonary vein reconnection. A total of 29 applications were delivered to the RSPV and 16 to the RIPV, including 13 and 16 applications to the RSPV ostium and antrum, respectively. A chest radiograph obtained the day after the procedure demonstrated elevation of the right hemidiaphragm, consistent with right phrenic nerve palsy (Figure 2A,B). Because the patient remained asymptomatic during hospitalization, she was discharged without additional intervention. At the 1‐month follow‐up, sinus rhythm was maintained, with a reduction in the cardiac silhouette on chest radiography and a decrease in the BNP level from 169.2 to 59.0 pg/mL. However, she complained of exertional dyspnea during prolonged walking. Chest radiography continued to demonstrate elevation of the right hemidiaphragm (Figure 3A), and spirometry revealed both restrictive and obstructive ventilatory defects. At the 3‐month follow‐up, both the elevated right hemidiaphragm and exertional dyspnea persisted. At the 6‐month follow‐up, chest radiography demonstrated normalization of the right hemidiaphragm position (Figure 3B). The patient's exertional dyspnea had markedly improved, and repeat spirometry demonstrated marked improvement in both restrictive and obstructive ventilatory abnormalities (Table 1). The anatomical courses of the right phrenic nerve and pericardiacophrenic artery in this patient are shown in Figure 4.

**FIGURE 1:**
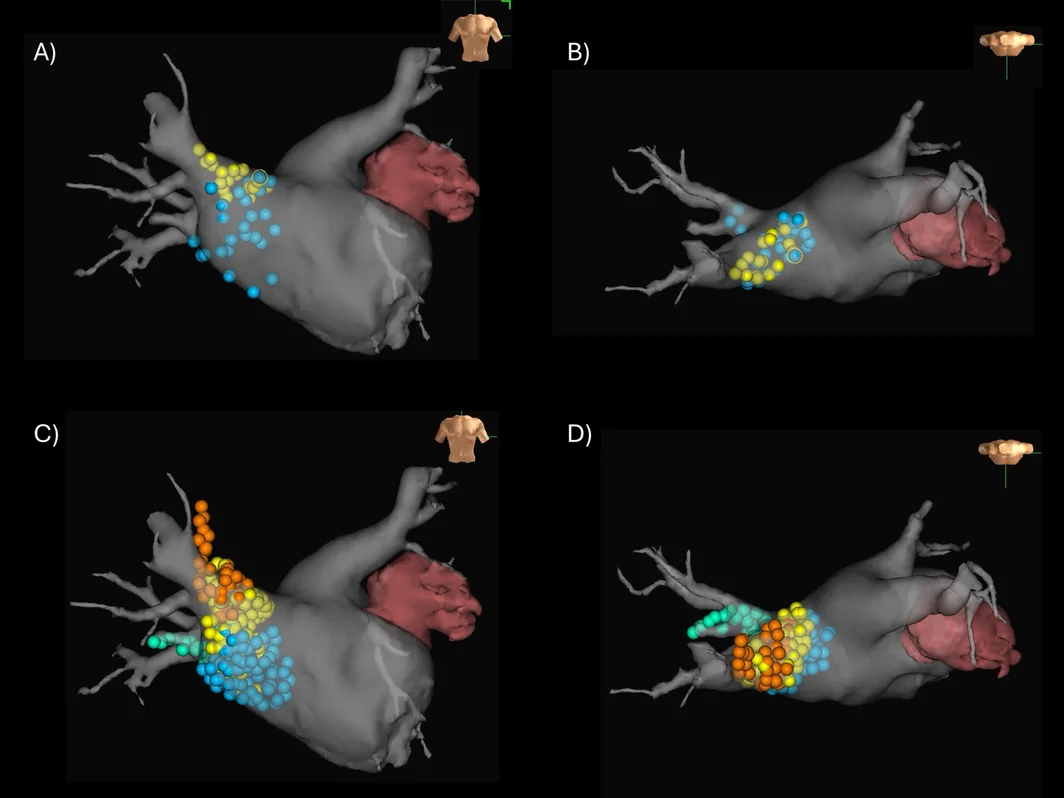
Application sites of the PulseSelect catheter in the right superior pulmonary vein (RSPV) and right inferior pulmonary vein (RIPV). Applications delivered to the RSPV ostium are indicated in orange, those to the RSPV antrum in yellow, those to the RIPV ostium in light green, and those to the RIPV antrum in sky blue. (A, B) show only the fifth electrode tagged, whereas (C, D) show all electrodes of the PulseSelect catheter tagged. Note that tagging all electrodes reveals applications extending further distally into the pulmonary veins (PVs). Upper left: Anteroposterior (AP) view. Upper right: Superior view. Lower left: Right anterior oblique (RAO) view. Lower right: Right cranial view. The right atrium is displayed in the lower panels.

**FIGURE 2:**
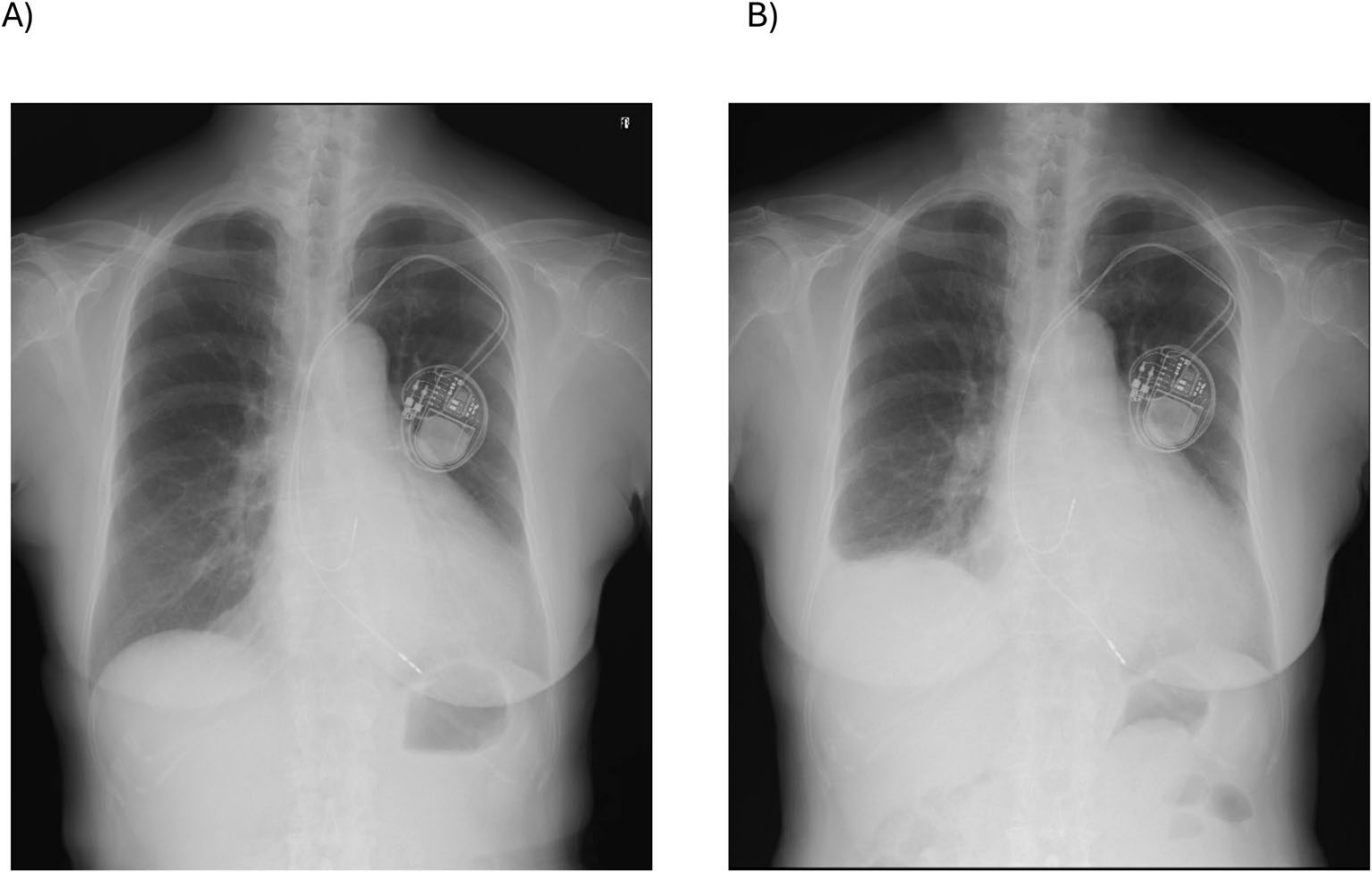
Chest radiographs obtained before catheter ablation (A) and on the day after the procedure (B). Elevation of the right hemidiaphragm, consistent with right phrenic nerve palsy, is evident on the post‐procedural chest radiograph.

**FIGURE 3:**
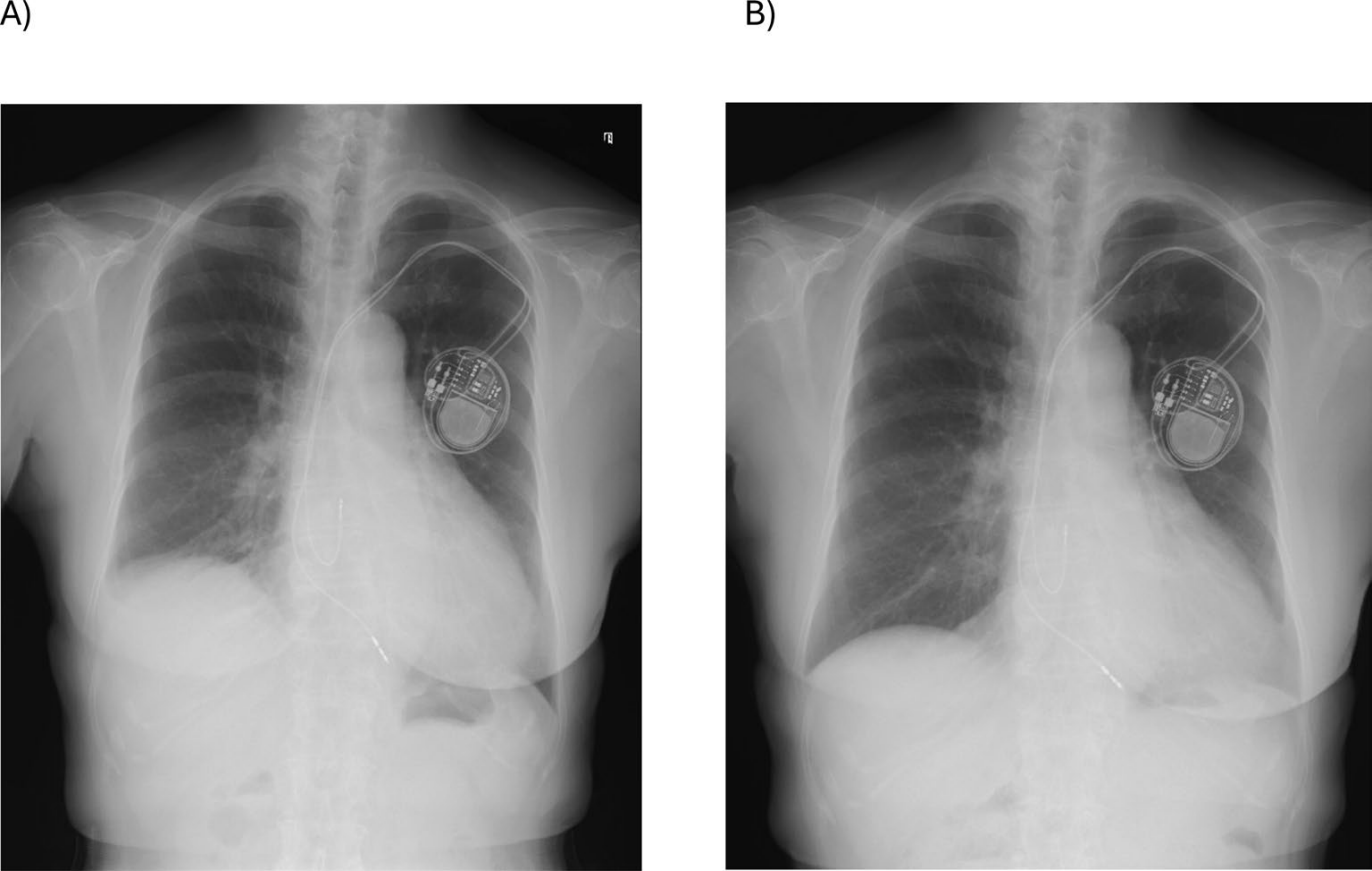
Chest radiograph obtained 1 month after catheter ablation (A), demonstrating persistent elevation of the right hemidiaphragm. Despite persistent diaphragmatic elevation, the cardiothoracic ratio had slightly decreased compared with the pre‐ablation chest radiograph. Chest radiograph obtained 6 months after catheter ablation (B), demonstrating normalization of the right hemidiaphragm position. Exertional dyspnea had also resolved.

**TABLE 1:** Serial pulmonary function test results before and after catheter ablation.

	VC (L) (%VC)	FEV1 (%FEV1)	FEV1%
1 month after PFA	1.77 (82.7%)	1.16 (67.8%)	65.9 (89.3%)
6 month after PFA (after recovery)	2.13 L (99.5%)	1.5 L (87.7%)	70.8 (95.8%)

**FIGURE 4:**
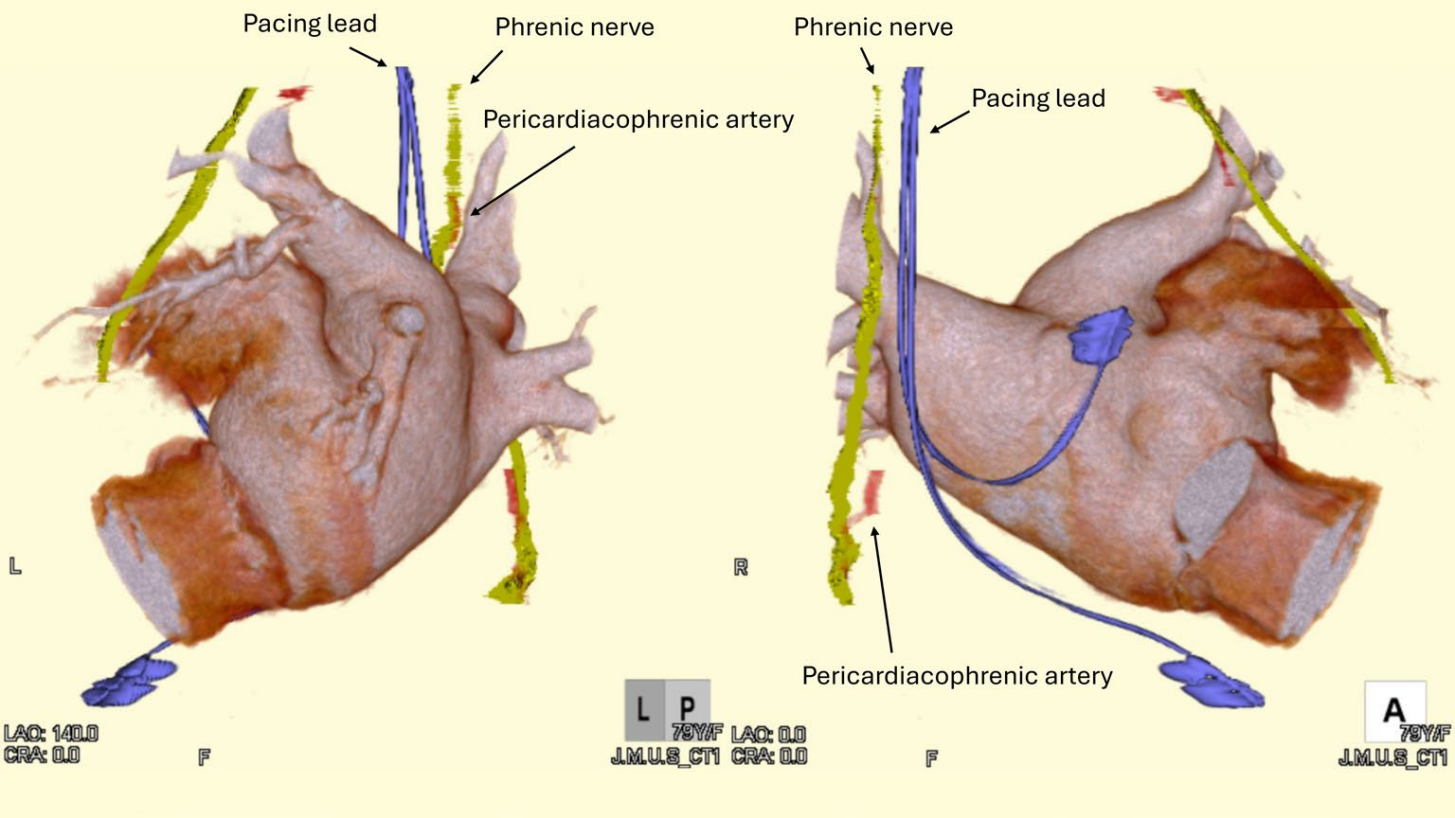
The image of the course of this patient's phrenic nerve and pericardiacophrenic artery is described.

The present case documents recovery from persistent phrenic nerve palsy approximately 6 months after PFA. To the best of our knowledge, this is the first published report describing recovery from persistent PFA‐related phrenic nerve palsy. Phrenic nerve injury is considered an extremely rare complication of PFA [1]. Nevertheless, recent reports have described persistent phrenic nerve palsy following AF ablation using PFA [2]. Although the irreversible electroporation (IRE) threshold of the human phrenic nerve has not been established, preclinical studies have reported a threshold of approximately 3800 V/cm for the sciatic nerve, a peripheral motor nerve considered physiologically similar to the phrenic nerve. This value is substantially higher than the electric field strength generated by the PulseSelect system (1600 V/cm). However, transient and clinically apparent phrenic nerve injury have been reported following PFA [3]. These observations suggest that electric field strength alone may not fully explain the occurrence of phrenic nerve injury.

The mechanisms underlying persistent phrenic nerve palsy after PFA remain unclear. PFA induces IRE in myocardial tissue and is generally considered to have relative tissue selectivity because cardiomyocytes have a lower threshold for IRE than many non‐cardiac tissues, including neural tissue. However, experimental studies have demonstrated structural neural injury following exposure of peripheral nerves to IRE, including axonal swelling, fragmentation or loss, Schwann cell proliferation, macrophage infiltration, and Wallerian degeneration [4]. These findings suggest that neural injury may occur along a spectrum rather than as an all‐or‐none phenomenon. One possible mechanism is cumulative neural injury resulting from repeated PFA applications. Although an individual application may not produce irreversible nerve injury, repeated applications near the same nerve could result in cumulative damage that exceeds its capacity for repair [5]. Such injury may affect the axonal membrane, nodes of Ranvier, myelin, or Schwann cells and ultimately impair nerve conduction, even without neuronal cell death. In addition, nodes of Ranvier may be particularly susceptible to electroporation, and temperature elevations have been reported during PFA despite its primarily nonthermal mechanism [6]. Thermal effects may therefore also contribute to nerve injury, particularly when multiple applications are delivered near the nerve. Nevertheless, these proposed mechanisms remain speculative and have not been directly demonstrated in clinical PFA‐related phrenic nerve palsy.

The spatial distribution of energy delivery may be particularly relevant to the PulseSelect system. Although the fifth electrode is commonly used as a fluoroscopic reference marker, energy is delivered from all electrodes. In our case, the application sites identified by tagging only the fifth electrode were not concentrated in the anterior RSPV. However, when the application sites of all electrodes were considered, a cluster of applications was observed in this region, and some applications had been delivered to more distal sites within the RSPV. Because the fifth electrode is oriented anteriorly, posterior tilting of the catheter to achieve adequate myocardial contact may position other electrodes more distally within the pulmonary vein. Consequently, the position of the fifth electrode alone may not accurately represent the spatial distribution of energy delivery from the entire catheter. This observation highlights the importance of considering the positions of all electrodes when assessing the potential risk of phrenic nerve injury.

Previous reports of persistent phrenic nerve palsy have also involved the PulseSelect system, raising the possibility of an association between this device and persistent phrenic nerve palsy [2]. Although PulseSelect delivers a relatively low electric field strength of 1600 V/cm, the cumulative tissue effects of multiple pulse trains and the intervals between applications remain incompletely understood. Furthermore, the catheter design may facilitate energy delivery at distal sites within the pulmonary veins, potentially increasing the risk of phrenic nerve injury. However, the available evidence is insufficient to establish that PulseSelect carries a higher risk of persistent phrenic nerve palsy than other PFA systems. Further studies are needed to determine whether catheter design, pulse characteristics, and application patterns contribute to this complication.

In our patient, 29 applications were delivered to the RSPV alone, including 13 at the ostium and 16 at the antrum. The total number of applications was 80, exceeding both our institutional average of 66 ± 15 applications (63 cases, October 2025–April 2026) and the mean of 67 ± 17 applications reported in a previous study [7]. The relatively high number of applications may have contributed to cumulative neural injury. However, the total number of applications alone may not fully explain the occurrence of phrenic nerve palsy. Repeated applications at the same or closely adjacent sites, together with their spatial distribution within the RSPV, may also have been important. Accordingly, attention should be paid not only to the total number of applications but also to their distribution and the position of all electrodes. Unnecessary applications should be avoided, and energy delivery deep within the pulmonary veins should be minimized when feasible. Repeated applications at the same or closely adjacent sites should also be minimized, particularly when the phrenic nerve is close to the target region. These considerations may be especially important when the anatomical relationship between the right phrenic nerve and the right‐sided pulmonary veins increases the potential for nerve injury.

Anatomical factors may also influence susceptibility to phrenic nerve injury. Persistent phrenic nerve palsy has been reported following cryoballoon ablation, particularly during right‐sided PVI [8]. A larger left atrial or pulmonary vein antral anatomy may result in deeper balloon positioning, bringing the ablation site closer to the phrenic nerve. Although the mechanism differs from that of PFA, a similar anatomical consideration may apply. A short distance between the right phrenic nerve and the right‐sided pulmonary veins, particularly the RSPV, may increase susceptibility to injury. Our patient developed symptomatic phrenic nerve palsy. A previous report described an elderly woman with a BMI of 28.6 kg/m^2^ who developed symptomatic palsy after cryoballoon ablation and required thoracoscopic diaphragmatic plication [9]. In our patient, thoracoscopic diaphragmatic plication would also have been considered had the symptoms persisted. Pott et al. reported that female sex and lower BMI were independent risk factors for persistent phrenic nerve palsy after cryoballoon ablation [10]. They proposed that smaller thoracic dimensions and less insulating adipose tissue may increase the proximity between the right phrenic nerve and the right‐sided pulmonary veins. Although these findings cannot be directly extrapolated to PFA, they suggest that patient‐specific anatomy may influence susceptibility to phrenic nerve injury. Previous reports of persistent PFA‐related phrenic nerve palsy also involved elderly women [2]. Therefore, particular attention may be warranted in patients whose anatomical characteristics may place the right phrenic nerve close to the RSPV.

Catheter ablation in our patient was performed under general anesthesia with neuromuscular blockade. Although general anesthesia is an established approach for AF ablation, deep sedation may allow earlier detection of phrenic nerve dysfunction by preserving the patient's ability to report symptoms. However, it remains unclear whether either anesthetic strategy reduces the risk of nerve injury. Regardless of the anesthesia method, careful catheter positioning and an appropriate application strategy are important. In addition, chest radiography on the day after the procedure may facilitate early detection of phrenic nerve palsy, including in patients who remain asymptomatic immediately after ablation.

Our patient had a permanent pacemaker with leads in the right atrial appendage and right ventricular apex. Although PFA can cause electromagnetic interference with pacing systems, there is no established evidence that pacemakers or their leads increase the risk of phrenic nerve injury. In our patient, the leads were also distant from the PFA sites and the presumed course of the right phrenic nerve. Therefore, the pacemaker was unlikely to have contributed substantially to the phrenic nerve palsy.

The mechanisms underlying recovery from PFA‐related phrenic nerve palsy also remain uncertain. Transient palsy may reflect reversible impairment of axonal excitability or conduction, whereas prolonged dysfunction followed by recovery may indicate more substantial but reversible axonal injury. Experimental studies have demonstrated axonal swelling and degeneration after IRE, followed by macrophage infiltration and Schwann cell proliferation, suggesting the potential for neural repair [4]. However, whether these processes contribute to recovery after clinical PFA remains unknown. The recovery observed in our patient may therefore reflect the resolution of reversible neural dysfunction or repair following structural axonal injury rather than true nerve regeneration.

Most published reports of phrenic nerve palsy after AF ablation involve radiofrequency or cryoballoon ablation, whereas data on PFA‐related phrenic nerve palsy remain limited. More than half of patients with phrenic nerve palsy after catheter ablation recover within 6 months [11]. The recovery observed at approximately 6 months in our patient is consistent with this previously reported clinical course. Nevertheless, PFA systems differ in catheter design, waveform, and electric field characteristics, and their associated risks and clinical courses may therefore differ. Larger studies are needed to clarify the incidence, mechanisms, natural history, and prognosis of PFA‐related phrenic nerve palsy.

## Funding

The authors have nothing to report.

## Conflicts of Interest

The authors declare no conflicts of interest.

## Data Availability

The data that support the findings of this study are available on request from the corresponding author. The data are not publicly available due to privacy or ethical restrictions.
